# Neuroblastoma Masquerading as a Septic Hip Infection in a Three-Year-Old

**DOI:** 10.7759/cureus.36350

**Published:** 2023-03-19

**Authors:** Joseph D Lynch, Patrick J Tomboc

**Affiliations:** 1 Pediatric Hospital Medicine, West Virginia University School of Medicine, Morgantown, USA; 2 Pediatric Hematology-Oncology, West Virginia University School of Medicine, Morgantown, USA

**Keywords:** chatgpt improved case report, pediatric clinical conundrum, neuroblastoma metastases, pediatric septic joint, pediatric hematology-oncology, pediatric hospital medicine, inpatient pediatrics, neuroblastoma, chatgpt

## Abstract

Metastatic neuroblastoma to the bone and septic joint shares the same incidence in age and clinical symptomology. Here we discuss a three-year-old male who presented with anemia, persistent hip pain, and a refusal to bear weight. A thorough evaluation based on a broad differential diagnosis allowed for an expedient diagnosis of metastatic neuroblastoma. The timely diagnosis allowed for rapid enrolment in a children’s oncology group (COG) clinical trial for advanced neuroblastoma. The patient tolerated the therapy without adverse events and remains in remission.

## Introduction

This case report is based on a poster presentation of the same case presented at the Pediatric Hospital Medicine Conference in 2021 by Dr. Lynch. Both septic joint infection and neuroblastoma are common diagnoses in the toddler age group [1,2]. Their symptomatology does not widely overlap in most cases [1,2]. Here we present a unique case of an advanced stage IV neuroblastoma with bone metastases masquerading as a septic hip in a three-year-old child. Initial evaluation was consistent with a septic hip, but persistent pain, refusal to bear weight, and laboratory abnormalities despite washout and antibiotics eventually led to bone marrow aspiration and the diagnosis of neuroblastoma. Once correctly diagnosed, the child began to improve with chemotherapy.

## Case presentation

A three-year-old child was transferred to our facility with a presumptive diagnosis of a septic right hip. He had a three-week history of the right knee and hip pain, fevers, fatigue, and the inability to bear weight on his right leg. He had presented multiple times to outside emergency departments (ED). Ten days before transfer, he had a traumatic fall on his right knee and was seen and discharged without intervention. Seven days before admission, he was seen again and prescribed amoxicillin for seven days, which he completed. He failed antibiotic therapy, spiked a fever, and experienced progressive right knee and hip pain throughout.

At the referring ED, he was febrile, hypotensive (75/40 mm Hg), and had multiple abnormal labs (elevated CRP 179 mg/L, LDH 598 units/L, hypoalbuminemia of 2.8 g/dL, and anemia of 8.1 g/dL). His blood cell lines were normal, with a white blood cell (WBC) count of 7.4 x 103 /uL, a platelet (PLT) count of 271 x 103 /uL, hemoglobin of 11.1 x 103 /uL, and a hematocrit of 32.5%. On arrival at our facility, he was febrile at 38.8oC, tachycardic at 137, and his hypotension had resolved after fluid resuscitation before transport. He was markedly irritable and screamed in pain with any manipulation of his right leg. He could not bear weight on the right leg secondary to pain. There is no joint erythema, swelling, or effusion appreciated in the knee or hip. At this point, the patient satisfies 3 of the 4 Kocher criteria (elevated ESR, temperature > 38.5oC, and non-weight-bearing status), conferring a 93% probability of septic arthritis. The remainder of his physical examination is unremarkable. Grandmother denies other symptoms over the last month, including rashes, nausea, vomiting, weight loss, abdominal pain, diarrhea, conjunctivitis, or other illnesses. She denies any sick contacts and has not discovered any ticks on the patient in the last six weeks.

**Figure 1:**
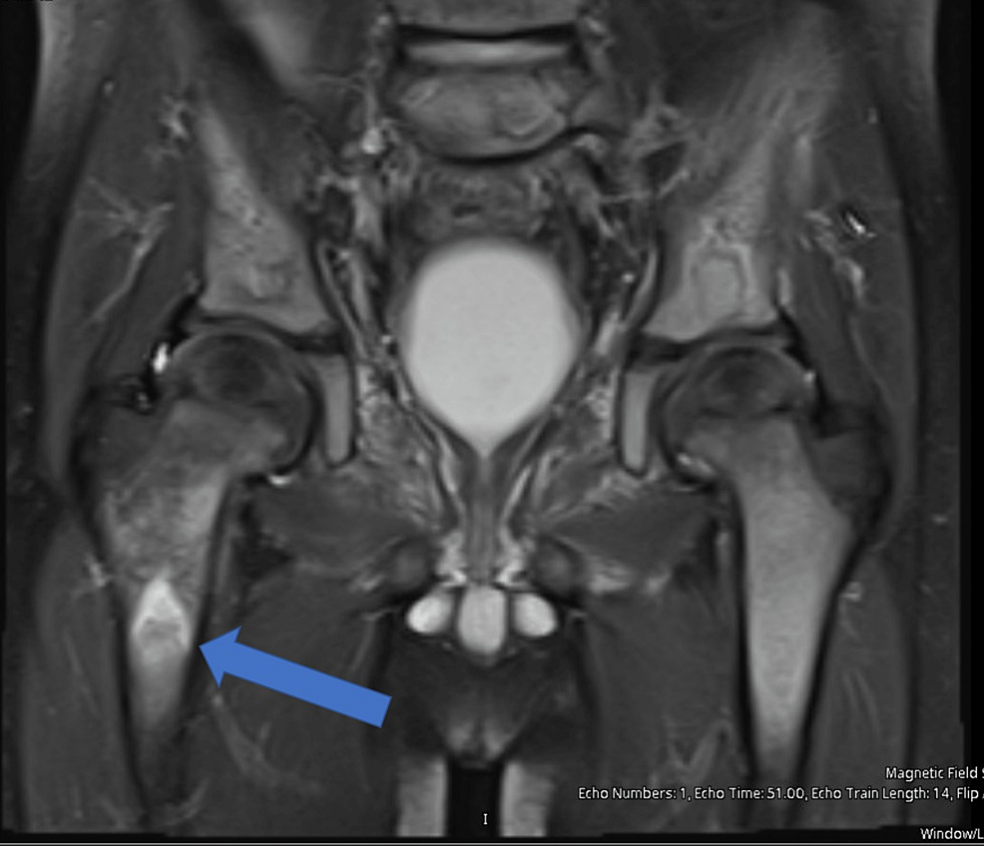
T2 weighted image showing increased marrow enhancement of the right trochanteric area (indicated by the blue arrow) as compared to the left. T2: Transverse relaxation time

**Figure 2:**
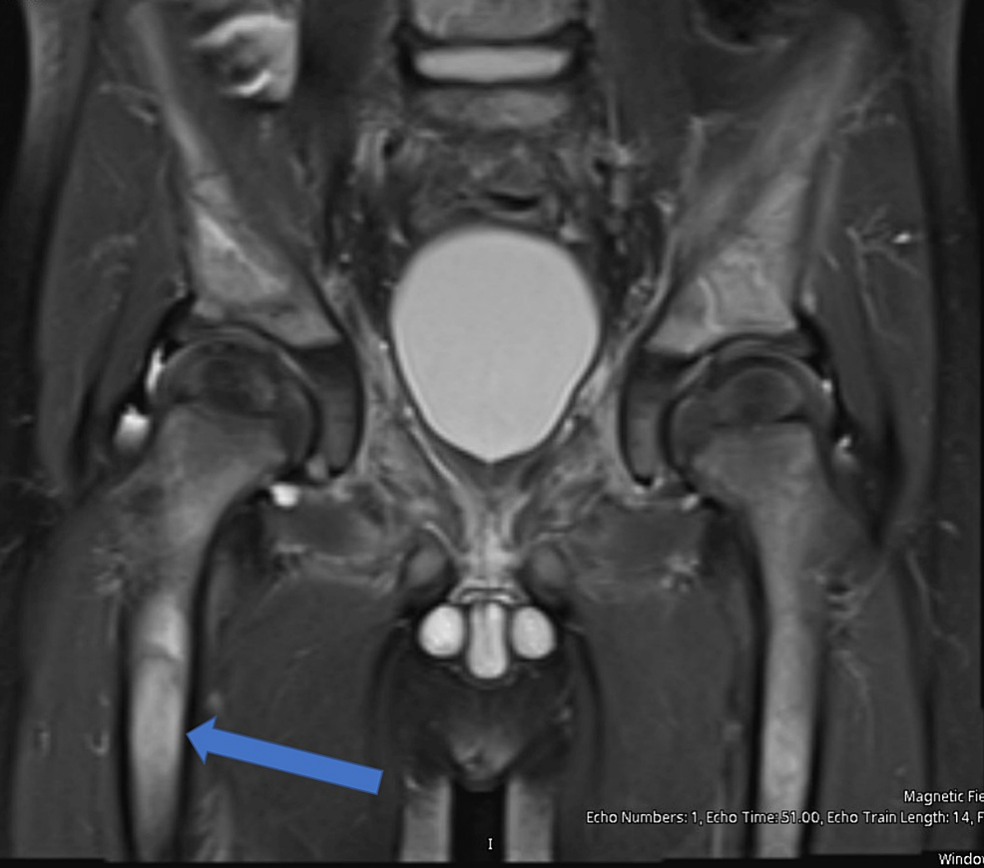
MRI of the right hip/femur demonstrating increased marrow signal on T2. The blue arrow is indicative of the increased signal seen in the more distal femur area as compared to Figure 1. T2: Transverse relaxation time, MRI: Magnetic resonance imaging

The patient was transfused 15 mL/kg of packed red blood cells (PRBC) for symptomatic anemia. Given the triad of fever, inability to bear weight, and elevated inflammatory makers, the patient underwent a sedated MRI of the right hip and knee. MRI showed an abnormal marrow signal from the right femoral neck through the subtrochanteric region, and a small hip effusion was felt to favor osteomyelitis over possible malignancy (Figures 1, 2). This prompted surgical washout and culture of the right hip by orthopedics. Reportedly, there was minimal soft tissue inflammation when the hip was open, making osteomyelitis unlikely; however, there was concern for a bony infarct. The patient was started on vancomycin and cefazolin postoperatively. 

Over the next four days, the patient remained hemodynamically stable following the PRBC transfusion. However, his pain, fatigue, and fevers only improved marginally with all remaining present. His inflammatory markers remained elevated with the following trends (from least to most recent): C-reactive protein (CRP) (227 to 219 to 175 to 209 mg/L) and erythrocyte sedimentation rate (ESR) (145 to 95 to 92 to 104 mm/hr). His white blood cells (WBC) and platelet count (PLT) continued to be surprisingly normal despite the large degree of inflammation within his body. The cultures from his knee and hip did not grow anything.

**Figure 3:**
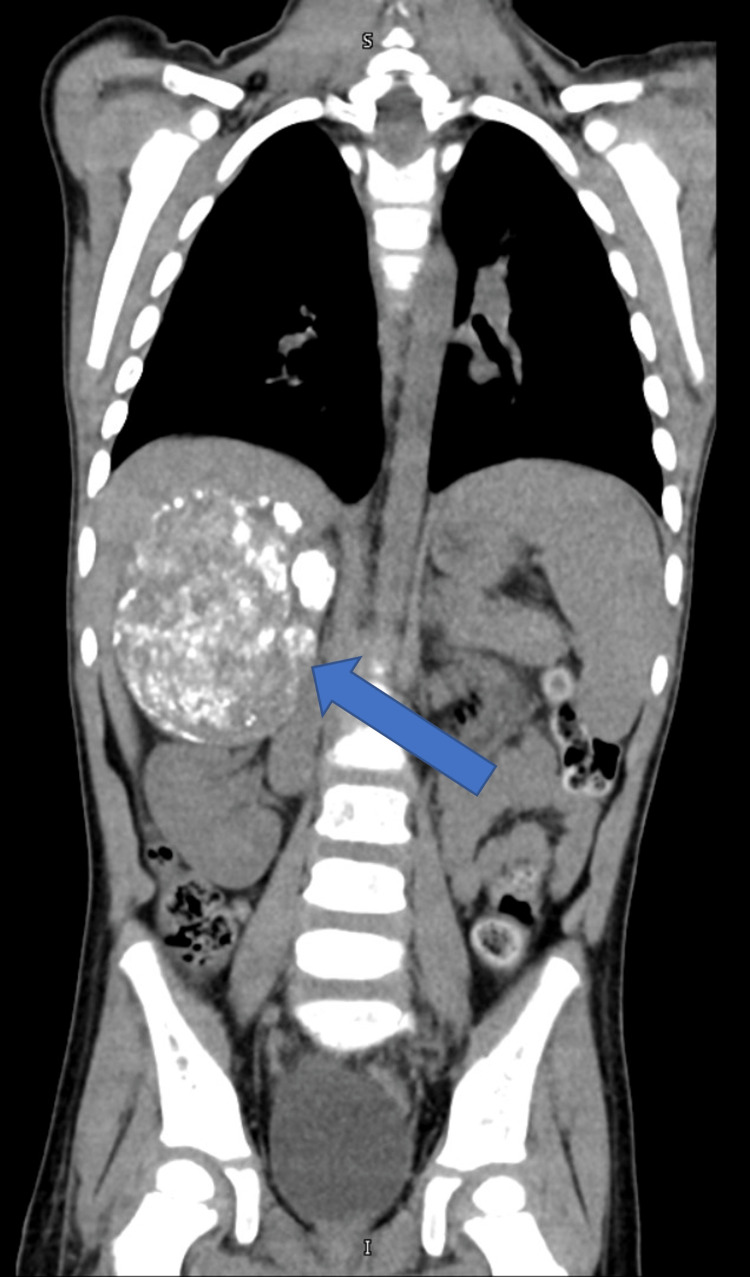
A large, well-defined, calcified, heterogenous mass arising from the right adrenal fossa, measuring consistent with a neuroblastoma. The mass is indicated by a blue arrow and can be seen displacing the right kidney downward.

On hospital day five, a bone marrow aspiration was performed by the Heme/Onc service, which showed poorly differentiated neuroblastoma. This was consistent with the now resulted surgical pathology. Urine vanillylmandelic acid (VMA) (121.9 mg/g Cr, normal <16.0 mg/g Cr) and homovanillic acid (HVA) (161.9 mg/g Cr, normal < 25.0 mg/g Cr) were sent and were significantly elevated. A meta-iodo-benzyl-gaunidine (MIBG) computed tomography (CT) scan was performed and showed non-avid disease and a large (6.4 x 6.3 x 8.1 cm) poorly differentiated neuroblastoma arising from the right adrenal fossa (Figure 3). The patient was initiated on chemotherapy, COG ABNL 1531 (cyclophosphamide/topotecan), nine days after transfer. The patient was discharged after 24 days.

To achieve remission, the patient received a total of five cycles of chemotherapy. MIBG therapy was given after the third cycle, and local surgical control occurred following the fourth cycle of therapy. After completion of the fifth cycle of chemotherapy, the patient received Busulfan/Melphalan with a hematopoietic stem cell transplant (HSCT). He then received local radiation therapy (21.6 gray in 12 fractions). Post-consolidation therapy consisted of six cycles utilizing Dinutuximab, GSCF, and isotretinoin. He completed therapy in December of 2022 and remains in remission at the time of this publication. 

## Discussion

The purview of a pediatric oncologist’s world, which is primarily clinical, is divided into one-third of leukemia/lymphoma, one-third of the central nervous system (CNS) neoplasms, and one-third of all other pediatric cancers [1]. B-Cell acute lymphoblastic leukemia (ALL) is the most common pediatric cancer and, thus, the most common diagnosis seen by pediatric oncologists. While neuroblastoma is the most common non-CNS solid neoplasm found in children, it accounts for only 6%-8% of all pediatric cancers [1]. A three-year-old presenting with cytopenia and leg pain is a common presentation for a patient with B-cell ALL. Generating a comprehensive differential diagnosis that included metastatic disease likely shortened this patient’s time to diagnosis.

Bone pain, fever, and an inability to bear weight should trigger the generalist pediatrician to consider other conditions that must be immediately acted upon. These conditions include but are not limited to osteomyelitis, leukemia, lymphoma, septic joint infection, or other cancerous processes. The most frequently encountered condition in the pediatric hospitalist world would be septic joint, affecting 4-5 cases per 100,000 children per year [2]. Children affected by septic joint infection are in the same general age range (2 to 5 years old) and have overlapping symptoms as those in the early peak of neuroblastoma. Overlap of these conditions makes sense, as neuroblastoma often metastasizes to the bone and can mimic septic joint infections.

Neuroblastoma has an incidence of 1.1 to 1.3 cases per 100,000 children aged 15 or younger [3]. It is the most common extracranial solid tumor in children. Around 50% of patients diagnosed with neuroblastoma will have evidence of metastasis when they are initially diagnosed [4,5]. The most common site of metastasis is the bone [6], which brings into focus our patient’s presentation. Commonly affected bones include the pelvis, skull, face, and extremities [6]. Providers should be aware of the common patterns of neuroblastoma metastasis and how it may mimic other diseases, such as septic joint infection.

## Conclusions

In summary, our case highlights a three-year-old male who presented with anemia and bone pain and was found to have metastatic neuroblastoma (consider PET/CT refer). This patient’s presentation is consistent with the most common pediatric cancer, ALL, but due to a broad differential diagnosis and workup, the correct diagnosis was found without delay. Due to the timeliness of his diagnosis, he was enrolled in a children’s oncology group study, which provided the advanced multimodal therapy necessary to place him in a durable remission.

## Figures and Tables

**Figure 4:**
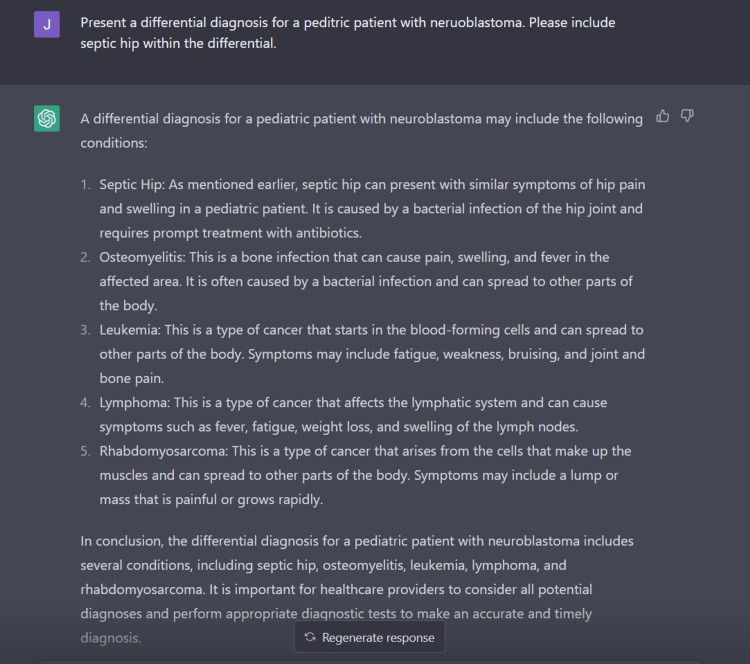
Screenshot of ChatGPT's response to being asked to provide a differential diagnosis for a pediatric patient with neuroblastoma, including septic hip

**Figure 5:**
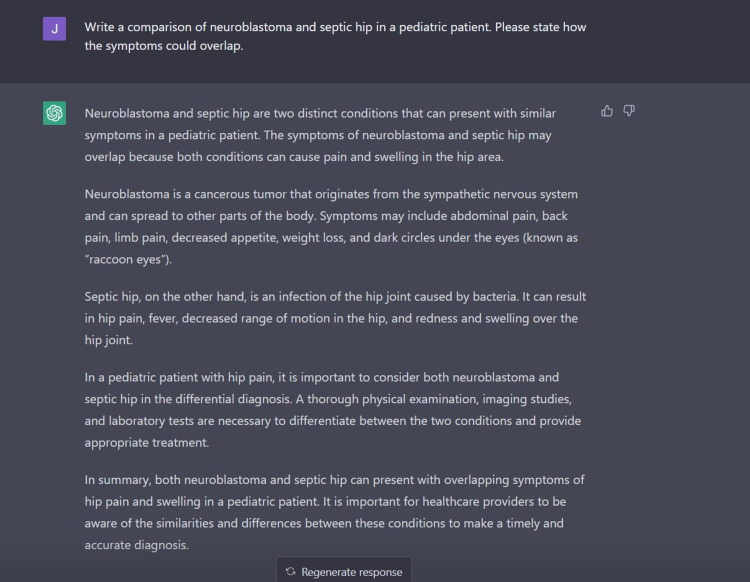
ChatGPT's comparison of neuroblastoma and septic hip

**Figure 6:**
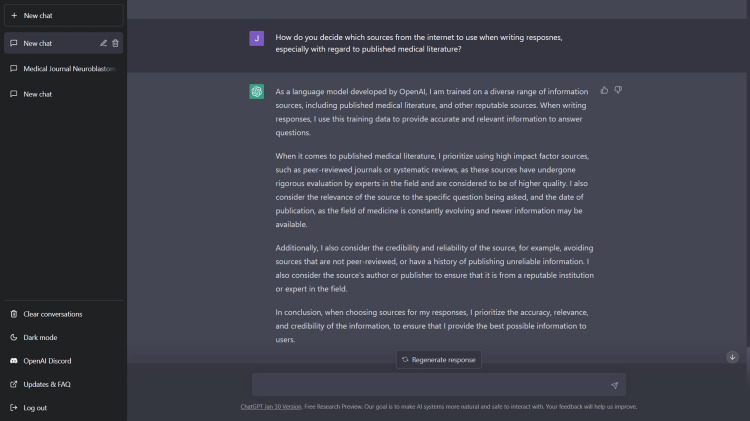
ChatGPT's response to how it decides what medical literature to source from

## Appendices

Figure 4 is a differential generated by ChatGPT. It provided a reasonable comparison of different conditions that could fit the symptoms of neuroblastoma or septic hip. It went into specific details that we did not feel were necessary to the general message of the paper.

The comparison of neuroblastoma and septic hip is well written by ChatGPT (Figure 5) and helped guide our discussion section. It fails to mention the reasons why neuroblastoma could cause hip pain, which is an important idea, but otherwise connects the two diseases well. As in other cases when we have used ChatGPT, we have found that the AI tends to restate information in slightly different ways that make the information appear somewhat superficial as compared to what would be written by a subject matter expert.

We were very curious about how ChatGPT sourced its responses from the medical literature. In Figure 6, ChatGPT reveals that it pulls data up to 2021 and only from peer-reviewed sources. It did not make any references to impact factors or journal reputation but did allude to experts being of “higher quality.”

Overall, we found ChatGPT to be a useful tool in helping with the authorship of this case report. It lacked some important details and information about where the data was being pulled from. It was able to bring information together, especially when comparing disease states that would have taken human authors longer lengths of time. It will be interesting to see how the medical literature changes as AI becomes more sophisticated.
